# 1-[2-(2,6-Dichloro­benz­yloxy)-2-(2-fur­yl)eth­yl]-1*H*-1,2,4-triazole

**DOI:** 10.1107/S1600536809052568

**Published:** 2009-12-12

**Authors:** Özden Özel Güven, Hakan Tahtacı, Simon J. Coles, Tuncer Hökelek

**Affiliations:** a Department of Chemistry, Zonguldak Karaelmas University, 67100 Zonguldak, Turkey; b Department of Chemistry, Southampton University, Southampton SO17 1BJ, England; c Department of Physics, Hacettepe University, 06800 Beytepe, Ankara, Turkey

## Abstract

In the mol­ecule of the title compound, C_15_H_13_Cl_2_N_3_O_2_, the triazole ring is oriented at dihedral angles of 2.54 (13) and 44.43 (12)°, respectively with respect to the furan and dichloro­benzene rings. The dihedral angle between the dichloro­benzene and furan rings is 46.75 (12)°. In the crystal structure, inter­molecular C—H⋯O hydrogen bonds link the mol­ecules into centrosymmetric dimers and π–π contacts between dichloro­benzene rings [centroid–centroid distance = 3.583 (2) Å] may further stabilize the structure. Inter­molecular C—H⋯π contacts between the triazole and furan rings also occur.

## Related literature

For general background to anti­fungal agents, see: Caira *et al.* (2004 ▶); Godefroi *et al.* (1969 ▶); Özel Güven *et al.* (2007*a* ▶,*b* ▶); Paulvannan *et al.* (2001 ▶); Peeters *et al.* (1996 ▶); Wahbi *et al.* (1995 ▶). For related structures, see: Freer *et al.* (1986 ▶); Özel Güven *et al.* (2008*a* ▶,*b* ▶,*c* ▶,*d* ▶,*e* ▶,*f* ▶); Özel Güven *et al.* (2009 ▶); Peeters *et al.* (1979 ▶).
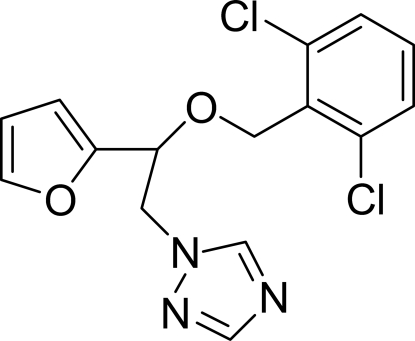


## Experimental

### 

#### Crystal data

C_15_H_13_Cl_2_N_3_O_2_
*M* *_r_* = 338.18
Monoclinic, 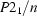

*a* = 10.5853 (3) Å
*b* = 12.4960 (2) Å
*c* = 12.5850 (3) Å
β = 114.455 (1)°
*V* = 1515.32 (6) Å^3^
*Z* = 4
Mo *K*α radiation
μ = 0.44 mm^−1^
*T* = 120 K
0.40 × 0.40 × 0.10 mm

#### Data collection

Bruker–Nonius KappaCCD diffractometer
Absorption correction: multi-scan (*SADABS*; Sheldrick, 2007 ▶) *T*_min_ = 0.837, *T*_max_ = 0.955
33067 measured reflections
3438 independent reflections
2775 reflections with *I* > 2σ(*I*)
*R*_int_ = 0.058

#### Refinement

*R*[*F* ^2^ > 2σ(*F* ^2^)] = 0.067
*wR*(*F* ^2^) = 0.180
*S* = 1.04
3438 reflections
200 parameters
H-atom parameters constrained
Δρ_max_ = 1.20 e Å^−3^
Δρ_min_ = −0.76 e Å^−3^

### 

Data collection: *COLLECT* (Nonius, 1998 ▶); cell refinement: *DENZO* (Otwinowski & Minor, 1997 ▶) and *COLLECT*; data reduction: *DENZO* and *COLLECT*; program(s) used to solve structure: *SHELXS97* (Sheldrick, 2008 ▶); program(s) used to refine structure: *SHELXL97* (Sheldrick, 2008 ▶); molecular graphics: *ORTEP-3 for Windows* (Farrugia, 1997 ▶) and *PLATON* (Spek, 2009 ▶); software used to prepare material for publication: *WinGX* (Farrugia, 1999 ▶) and *PLATON*.

## Supplementary Material

Crystal structure: contains datablocks I, global. DOI: 10.1107/S1600536809052568/xu2704sup1.cif

Structure factors: contains datablocks I. DOI: 10.1107/S1600536809052568/xu2704Isup2.hkl

Additional supplementary materials:  crystallographic information; 3D view; checkCIF report

## Figures and Tables

**Table 1:** Hydrogen-bond geometry (Å, °)

*D*—H⋯*A*	*D*—H	H⋯*A*	*D*⋯*A*	*D*—H⋯*A*
C2—H2⋯O1^i^	0.93	2.44	3.363 (3)	173
C9—H9*B*⋯Cl2	0.97	2.62	3.109 (3)	112
C1—H1⋯*Cg*2^ii^	0.93	2.79	3.488 (4)	133
C7—H7⋯*Cg*1^iii^	0.93	2.93	3.570 (4)	127

Symmetry codes: (i) 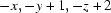; (ii) 
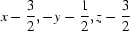
; (iii) 
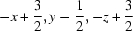
. *Cg*1 and *Cg*2 are the centroids of the N1–N3/C1/C2 and O2/C5–C8 rings, respectively.

**Fig. 1.**
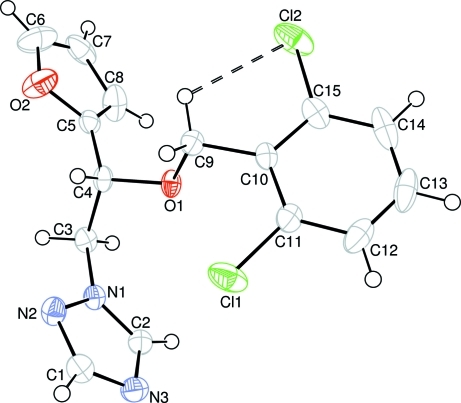
The molecular structure of the title compound with the atom-numbering scheme. Displacement ellipsoids are drawn at the 50% probability level. The dashed line indicates a hydrogen bond.

**Fig. 2.**
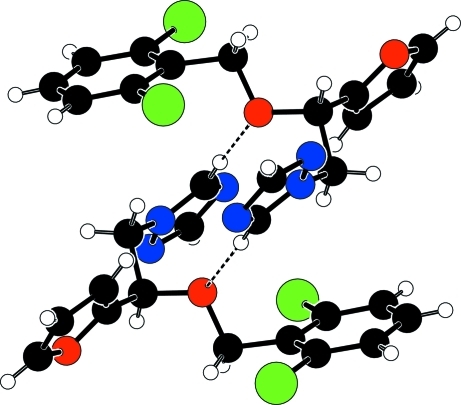
A partial packing diagram.

## supplementary crystallographic information

### Comment

In recent years, among antifungal agents, azole derivatives still have an important place in the class of systemic antifungal drugs. Some ether structures containing 1*H*-imidazole ring like micozanole, ecozanole and sulconazole have been synthesized and developed for clinical uses as antifungal agents (Godefroi *et al.*, 1969). The crystal structures of these ether derivatives like miconazole (Peeters *et al.*, 1979), econazole (Freer *et al.*, 1986) have been reported previously. Also, antifungal activity of aromatic ethers possessing 1*H*-1,2,4-triazole ring have been reported (Wahbi *et al.*, 1995). Itraconazole (Peeters *et al.*, 1996) and fluconazole (Caira *et al.*, 2004) are 1*H*-1,2,4-triazole ring containing azole derivatives. 1,2,4-Triazoles are biologically interesting molecules and their chemistry is receiving considerable attention due to antihypertensive, antifungal and antibacterial properties (Paulvannan *et al.*,  2001). Ether structures possessing 1*H*-benzimidazole ring have been reported to show antibacterial activity more than antifungal activity (Özel Güven *et al.*, 2007*a*,b). The crystal structures of 1*H*-benzimidazole ring containing ether derivatives (Özel Güven *et al.*, 2008*a*,b,c,d) and also,1*H*-1,2,4-triazole ring containing ether derivatives have been reported recently (Özel Güven *et al.*, 2008e,f; Özel Güven *et al.*, 2009). Now, we report herein the crystal structure of 2,6-dichloro- derivative of 1*H*-1,2,4-triazole and furyl rings containing ether structure.

In the molecule of the title compound (Fig. 1) the bond lengths and angles are generally within normal ranges. The planar triazole ring is oriented with respect to the furan and dichlorobenzene rings at dihedral angles of 2.54 (13)° and 44.43 (12)°, respectively. Atoms C3, C4 and C9 are -0.064 (3), 0.039 (3) and -0.073 (3) Å away from the planes of the triazole, furan and dichlorobenzene, respectively. So, they are nearly coplanar with the adjacent rings. The dichlorobenzene ring is oriented with respect to the furan ring at a dihedral angle of 46.75 (12)°. An intramolecular C—H···Cl hydrogen bond (Table 1) results in the formation of a five-membered ring (Cl2/H9B/C9/C10/C15) adopting envelope conformation with H9B atom displaced by 0.210 (1) Å from the plane of the other ring atoms.

In the crystal structure, intermolecular C-H···O hydrogen bonds (Table 1) link the molecules into centrosymmetric dimers (Fig. 2), in which they may be effective in the stabilization of the structure. The π–π contact between the dichlorobenzene rings, Cg3—Cg3^i^ [symmetry code: (i) -x, -y, 1 - z, where Cg3 is centroid of the ring (C10-C15)] may further stabilize the structure, with centroid-centroid distance of 3.583 (2) Å. Intermolecular C—H···π interactions (Table 1) are also observed between the triazole and furan rings.

### Experimental

The title compound was synthesized by the reaction of 1-(furan-2-yl)-2-(1*H*-1,2,4-triazol-1-yl)ethanol (unpublished results) with NaH and appropriate benzyl halide. To a solution of alcohol (500 mg, 2.791 mmol) in DMF (4 ml) was added NaH (112 mg, 2.791 mmol) in small fractions. The appropriate benzyl halide (669 mg, 2.791 mmol) was added dropwise. The mixture was stirred at room temperature for 3 h, and excess hydride was decomposed with methyl alcohol (5 ml). After evaporation to dryness under reduced pressure, the crude residue was suspended with water and extracted with methylene chloride. The organic layer was dried over anhydrous sodium sulfate and then evaporated to dryness. The crude residue was purified by chromatography on a silica-gel column using chloroform as eluent. Crystals suitable for X-ray analysis were obtained by the recrystallization of the ether from isopropanol solution (yield; 500 mg, 53%).

### Refinement

H atoms were positioned geometrically, with C–H = 0.93, 0.98 and 0.97 Å for aromatic, methine and methylene H, respectively, and constrained to ride on their parent atoms with *U*_iso_(H) = 1.2*U*_eq_(C).

### Crystal data

**Table:**

C_15_H_13_Cl_2_N_3_O_2_	*F*(000) = 696
*M**_r_* = 338.18	*D*_x_ = 1.482 Mg m^−^^3^
Monoclinic, *P*2_1_/*n*	Mo *K*α radiation, λ = 0.71073 Å
Hall symbol: -P 2yn	Cell parameters from 23020 reflections
*a* = 10.5853 (3) Å	θ = 2.9–27.5°
*b* = 12.4960 (2) Å	µ = 0.44 mm^−^^1^
*c* = 12.5850 (3) Å	*T* = 120 K
β = 114.455 (1)°	Plate, colorless
*V* = 1515.32 (6) Å^3^	0.40 × 0.40 × 0.10 mm
*Z* = 4	

### Data collection

**Table:**

Bruker–Nonius KappaCCD diffractometer	3438 independent reflections
Radiation source: fine-focus sealed tube	2775 reflections with *I* > 2σ(*I*)
graphite	*R*_int_ = 0.058
φ and ω scans	θ_max_ = 27.5°, θ_min_ = 3.3°
Absorption correction: multi-scan (*SADABS*; Sheldrick, 2007)	*h* = −13→13
*T*_min_ = 0.837, *T*_max_ = 0.955	*k* = −14→15
33067 measured reflections	*l* = −16→16

### Refinement

**Table:**

Refinement on *F*^2^	Secondary atom site location: difference Fourier map
Least-squares matrix: full	Hydrogen site location: inferred from neighbouring sites
*R*[*F*^2^ > 2σ(*F*^2^)] = 0.067	H-atom parameters constrained
*wR*(*F*^2^) = 0.180	*w* = 1/[σ^2^(*F*_o_^2^) + (0.0984*P*)^2^ + 1.9705*P*] where *P* = (*F*_o_^2^ + 2*F*_c_^2^)/3
*S* = 1.04	(Δ/σ)_max_ < 0.001
3438 reflections	Δρ_max_ = 1.20 e Å^−^^3^
200 parameters	Δρ_min_ = −0.76 e Å^−^^3^
0 restraints	Extinction correction: *SHELXL97* (Sheldrick, 2008), Fc^*^=kFc[1+0.001xFc^2^λ^3^/sin(2θ)]^-1/4^
Primary atom site location: structure-invariant direct methods	Extinction coefficient: 0.051 (5)

### Special details

**Table:**

Geometry. All esds (except the esd in the dihedral angle between two l.s. planes) are estimated using the full covariance matrix. The cell esds are taken into account individually in the estimation of esds in distances, angles and torsion angles; correlations between esds in cell parameters are only used when they are defined by crystal symmetry. An approximate (isotropic) treatment of cell esds is used for estimating esds involving l.s. planes.
Refinement. Refinement of F^2^ against ALL reflections. The weighted R-factor wR and goodness of fit S are based on F^2^, conventional R-factors R are based on F, with F set to zero for negative F^2^. The threshold expression of F^2^ > 2sigma(F^2^) is used only for calculating R-factors(gt) etc. and is not relevant to the choice of reflections for refinement. R-factors based on F^2^ are statistically about twice as large as those based on F, and R- factors based on ALL data will be even larger.

### Fractional atomic coordinates and isotropic or equivalent isotropic displacement parameters (Å^2^)

**Table:**

	*x*	*y*	*z*	*U*_iso_*/*U*_eq_	
Cl1	0.32066 (8)	0.66344 (7)	1.08482 (8)	0.0470 (3)	
Cl2	0.29005 (8)	0.31220 (7)	0.82358 (9)	0.0497 (3)	
O1	0.07950 (17)	0.54757 (14)	0.86960 (15)	0.0231 (4)	
O2	−0.1088 (3)	0.5914 (2)	0.5685 (2)	0.0483 (6)	
N1	−0.0576 (2)	0.71549 (17)	0.93071 (19)	0.0226 (5)	
N2	−0.0121 (2)	0.81516 (18)	0.9183 (2)	0.0284 (5)	
N3	0.0458 (2)	0.7770 (2)	1.1089 (2)	0.0298 (5)	
C1	0.0483 (3)	0.8475 (2)	1.0282 (3)	0.0300 (6)	
H1	0.0898	0.9144	1.0487	0.036*	
C2	−0.0207 (3)	0.6947 (2)	1.0435 (2)	0.0255 (5)	
H2	−0.0393	0.6309	1.0724	0.031*	
C3	−0.1260 (3)	0.6463 (2)	0.8301 (2)	0.0248 (5)	
H3A	−0.2023	0.6846	0.7706	0.030*	
H3B	−0.1638	0.5842	0.8530	0.030*	
C4	−0.0241 (2)	0.6098 (2)	0.7800 (2)	0.0228 (5)	
H4	0.0197	0.6727	0.7628	0.027*	
C5	−0.0952 (3)	0.5454 (2)	0.6705 (2)	0.0250 (5)	
C6	−0.1805 (4)	0.5198 (4)	0.4817 (3)	0.0582 (11)	
H6	−0.2048	0.5311	0.4026	0.070*	
C7	−0.2101 (3)	0.4328 (3)	0.5259 (3)	0.0457 (9)	
H7	−0.2564	0.3726	0.4847	0.055*	
C8	−0.1574 (3)	0.4484 (3)	0.6500 (3)	0.0429 (8)	
H8	−0.1646	0.4016	0.7047	0.051*	
C9	0.2081 (3)	0.5444 (2)	0.8579 (2)	0.0244 (5)	
H9A	0.2416	0.6164	0.8565	0.029*	
H9B	0.1961	0.5086	0.7859	0.029*	
C10	0.3091 (2)	0.4839 (2)	0.9611 (2)	0.0224 (5)	
C11	0.3640 (3)	0.5315 (2)	1.0718 (2)	0.0283 (6)	
C12	0.4517 (3)	0.4781 (3)	1.1713 (3)	0.0424 (8)	
H12	0.4849	0.5122	1.2435	0.051*	
C13	0.4890 (3)	0.3753 (3)	1.1629 (3)	0.0459 (9)	
H13	0.5469	0.3388	1.2297	0.055*	
C14	0.4416 (3)	0.3250 (2)	1.0558 (4)	0.0427 (9)	
H14	0.4695	0.2555	1.0500	0.051*	
C15	0.3507 (3)	0.3794 (2)	0.9559 (3)	0.0304 (6)	

### Atomic displacement parameters (Å^2^)

**Table:**

	*U*^11^	*U*^22^	*U*^33^	*U*^12^	*U*^13^	*U*^23^
Cl1	0.0272 (4)	0.0521 (5)	0.0535 (5)	0.0023 (3)	0.0085 (3)	−0.0273 (4)
Cl2	0.0340 (4)	0.0412 (5)	0.0711 (6)	−0.0067 (3)	0.0191 (4)	−0.0283 (4)
O1	0.0144 (8)	0.0260 (9)	0.0257 (9)	0.0012 (6)	0.0052 (7)	0.0068 (7)
O2	0.0633 (16)	0.0525 (15)	0.0306 (12)	−0.0134 (12)	0.0208 (11)	−0.0054 (10)
N1	0.0183 (10)	0.0227 (11)	0.0247 (11)	−0.0008 (8)	0.0069 (8)	0.0013 (8)
N2	0.0284 (12)	0.0243 (12)	0.0289 (12)	−0.0032 (9)	0.0082 (9)	0.0016 (9)
N3	0.0262 (11)	0.0356 (13)	0.0269 (12)	0.0010 (10)	0.0105 (9)	−0.0031 (9)
C1	0.0244 (13)	0.0283 (14)	0.0341 (15)	−0.0025 (10)	0.0089 (11)	−0.0028 (11)
C2	0.0228 (12)	0.0282 (13)	0.0282 (13)	0.0003 (10)	0.0131 (11)	0.0020 (10)
C3	0.0172 (11)	0.0250 (13)	0.0271 (13)	−0.0020 (9)	0.0041 (10)	−0.0012 (10)
C4	0.0168 (11)	0.0230 (12)	0.0233 (12)	0.0005 (9)	0.0032 (9)	0.0040 (9)
C5	0.0201 (12)	0.0264 (13)	0.0247 (13)	0.0046 (9)	0.0057 (10)	0.0000 (10)
C6	0.065 (3)	0.077 (3)	0.0307 (17)	−0.009 (2)	0.0176 (17)	−0.0201 (17)
C7	0.0299 (15)	0.0392 (18)	0.051 (2)	0.0080 (13)	−0.0004 (14)	−0.0203 (15)
C8	0.0333 (16)	0.0322 (16)	0.0470 (19)	−0.0052 (12)	0.0004 (14)	0.0062 (13)
C9	0.0177 (11)	0.0308 (13)	0.0254 (13)	0.0016 (9)	0.0096 (10)	0.0038 (10)
C10	0.0152 (11)	0.0240 (12)	0.0286 (13)	0.0001 (9)	0.0095 (10)	0.0050 (10)
C11	0.0165 (11)	0.0400 (15)	0.0289 (14)	0.0013 (10)	0.0098 (10)	0.0039 (11)
C12	0.0208 (13)	0.080 (3)	0.0261 (15)	0.0038 (14)	0.0098 (12)	0.0119 (15)
C13	0.0222 (14)	0.068 (2)	0.0461 (19)	0.0056 (14)	0.0131 (13)	0.0354 (17)
C14	0.0233 (14)	0.0281 (15)	0.080 (3)	0.0056 (11)	0.0243 (16)	0.0228 (15)
C15	0.0190 (12)	0.0248 (13)	0.0472 (17)	−0.0040 (10)	0.0134 (12)	−0.0008 (11)

### Geometric parameters (Å, °)

**Table:**

N1—N2	1.367 (3)	C7—C8	1.438 (5)
C1—N2	1.324 (4)	C7—H7	0.9300
C1—N3	1.352 (4)	C8—H8	0.9300
C1—H1	0.9300	C9—O1	1.429 (3)
C2—N3	1.323 (4)	C9—H9A	0.9700
C2—N1	1.333 (3)	C9—H9B	0.9700
C2—H2	0.9300	C10—C9	1.501 (3)
C3—N1	1.455 (3)	C10—C11	1.401 (4)
C3—H3A	0.9700	C10—C15	1.389 (4)
C3—H3B	0.9700	C11—Cl1	1.737 (3)
C4—O1	1.434 (3)	C11—C12	1.383 (4)
C4—C3	1.527 (4)	C12—C13	1.361 (6)
C4—C5	1.501 (4)	C12—H12	0.9300
C4—H4	0.9800	C13—C14	1.380 (6)
C5—O2	1.359 (4)	C13—H13	0.9300
C5—C8	1.352 (4)	C14—C15	1.403 (4)
C6—O2	1.373 (4)	C14—H14	0.9300
C6—C7	1.317 (6)	C15—Cl2	1.733 (3)
C6—H6	0.9300		
			
C9—O1—C4	112.62 (18)	C6—C7—C8	107.1 (3)
C5—O2—C6	106.5 (3)	C6—C7—H7	126.5
N2—N1—C3	120.9 (2)	C8—C7—H7	126.5
C2—N1—N2	109.7 (2)	C5—C8—C7	105.6 (3)
C2—N1—C3	129.2 (2)	C5—C8—H8	127.2
C1—N2—N1	101.5 (2)	C7—C8—H8	127.2
C2—N3—C1	102.1 (2)	O1—C9—C10	107.01 (19)
N2—C1—N3	115.7 (2)	O1—C9—H9A	110.3
N2—C1—H1	122.1	O1—C9—H9B	110.3
N3—C1—H1	122.1	C10—C9—H9A	110.3
N1—C2—H2	124.5	C10—C9—H9B	110.3
N3—C2—N1	110.9 (2)	H9A—C9—H9B	108.6
N3—C2—H2	124.5	C11—C10—C9	120.1 (2)
N1—C3—C4	110.8 (2)	C15—C10—C9	124.0 (2)
N1—C3—H3A	109.5	C15—C10—C11	115.9 (2)
N1—C3—H3B	109.5	C10—C11—Cl1	118.7 (2)
C4—C3—H3A	109.5	C12—C11—C10	122.9 (3)
C4—C3—H3B	109.5	C12—C11—Cl1	118.5 (3)
H3A—C3—H3B	108.1	C11—C12—H12	120.3
O1—C4—C3	106.1 (2)	C13—C12—C11	119.5 (3)
O1—C4—C5	111.1 (2)	C13—C12—H12	120.3
O1—C4—H4	109.3	C12—C13—C14	120.5 (3)
C3—C4—H4	109.3	C12—C13—H13	119.8
C5—C4—C3	111.5 (2)	C14—C13—H13	119.8
C5—C4—H4	109.3	C13—C14—C15	119.5 (3)
O2—C5—C4	117.2 (2)	C13—C14—H14	120.3
C8—C5—O2	110.3 (3)	C15—C14—H14	120.3
C8—C5—C4	132.5 (3)	C10—C15—Cl2	120.2 (2)
O2—C6—H6	124.7	C10—C15—C14	121.8 (3)
C7—C6—O2	110.6 (3)	C14—C15—Cl2	118.1 (2)
C7—C6—H6	124.7		
			
C2—N1—N2—C1	−1.0 (3)	C7—C6—O2—C5	−0.4 (4)
C3—N1—N2—C1	−176.9 (2)	O2—C6—C7—C8	1.3 (4)
N3—C1—N2—N1	0.4 (3)	C6—C7—C8—C5	−1.7 (4)
N2—C1—N3—C2	0.3 (3)	C10—C9—O1—C4	176.0 (2)
N3—C2—N1—N2	1.3 (3)	C11—C10—C9—O1	−73.1 (3)
N3—C2—N1—C3	176.7 (2)	C15—C10—C9—O1	104.8 (3)
N1—C2—N3—C1	−1.0 (3)	C9—C10—C11—Cl1	−3.8 (3)
C4—C3—N1—C2	−107.0 (3)	C9—C10—C11—C12	176.5 (2)
C4—C3—N1—N2	68.0 (3)	C15—C10—C11—Cl1	178.21 (19)
C3—C4—O1—C9	−156.0 (2)	C15—C10—C11—C12	−1.5 (4)
C5—C4—O1—C9	82.6 (3)	C9—C10—C15—Cl2	2.2 (3)
O1—C4—C3—N1	63.3 (3)	C9—C10—C15—C14	−177.5 (2)
C5—C4—C3—N1	−175.5 (2)	C11—C10—C15—Cl2	−179.85 (19)
O1—C4—C5—O2	−132.0 (2)	C11—C10—C15—C14	0.4 (4)
O1—C4—C5—C8	51.7 (4)	C10—C11—C12—C13	0.9 (4)
C3—C4—C5—O2	109.7 (3)	Cl1—C11—C12—C13	−178.8 (2)
C3—C4—C5—C8	−66.6 (4)	C11—C12—C13—C14	0.9 (4)
C4—C5—O2—C6	−177.9 (3)	C12—C13—C14—C15	−2.0 (4)
C8—C5—O2—C6	−0.8 (4)	C13—C14—C15—Cl2	−178.4 (2)
O2—C5—C8—C7	1.5 (4)	C13—C14—C15—C10	1.3 (4)
C4—C5—C8—C7	178.0 (3)		

### Hydrogen-bond geometry (Å, °)

**Table:**

Cg1 and Cg2 are the centroids of the N1–N3/C1/C2 and O2/C5–C8 rings, respectively.

**Table:**

*D*—H···*A*	*D*—H	H···*A*	*D*···*A*	*D*—H···*A*
C2—H2···O1^i^	0.93	2.44	3.363 (3)	173
C9—H9B···Cl2	0.97	2.62	3.109 (3)	112
C1—H1···Cg2^ii^	0.93	2.79	3.488 (4)	133
C7—H7···Cg1^iii^	0.93	2.93	3.570 (4)	127

Symmetry codes: (i) −*x*, −*y*+1, −*z*+2; (ii) *x*−3/2, −*y*−1/2, *z*−3/2; (iii) −*x*+3/2, *y*−1/2, −*z*+3/2.
